# The adverse health effects of punitive immigrant policies in the United States: A systematic review

**DOI:** 10.1371/journal.pone.0244054

**Published:** 2020-12-16

**Authors:** Nicholas A. Vernice, Nicola M. Pereira, Anson Wang, Michelle Demetres, Lisa V. Adams

**Affiliations:** 1 Center for Global Health Equity, Geisel School of Medicine at Dartmouth, Hanover, New Hampshire, United States of America; 2 Department of Medicine, Weill Cornell Medicine, New York, New York, United States of America; 3 Samuel J. Wood Library & C.V. Starr Biomedical Information Center, Weill Cornell Medicine, New York, New York, United States of America; Drexel University School of Public Health, UNITED STATES

## Abstract

**Background:**

Immigrants in the United States (US) today are facing a dynamic policy landscape. The Trump administration has threatened or curtailed access to basic services for 10.5 million undocumented immigrants currently in the US. We sought to examine the historical effects that punitive laws have had on health outcomes in US immigrant communities.

**Methods:**

In this systematic review, we searched the following databases from inception–May 2020 for original research articles with no language restrictions: Ovid MEDLINE, Ovid EMBASE, Cochrane Library (Wiley), Web of Science Core Collection (Clarivate), CINAHL (EBSCO), and Social Work Abstracts (Ovid). This study is registered with PROSPERO, CRD42019138817. Articles with cohort sizes >10 that directly evaluated the health-related effects of a punitive immigrant law or policy within the US were included.

**Findings:**

6,357 studies were screened for eligibility. Of these, 32 studies were selected for inclusion and qualitatively synthesized based upon four themes that appeared throughout our analysis: (1) impact on healthcare utilization, (2) impact on women’s and children’s health, (3) impact on mental health services, and (4) impact on public health. The impact of each law, policy, mandate, and directive since 1990 is briefly discussed, as are the limitations and risk of bias of each study.

**Interpretation:**

Many punitive immigrant policies have decreased immigrant access to and utilization of basic healthcare services, while instilling fear, confusion, and anxiety in these communities. The federal government should preserve and expand access for undocumented individuals without threat of deportation to improve health outcomes for US citizens and noncitizens.

## Introduction

Immigration is an irrefutable and foundational tenet of the American nation. The United States (US) continues to maintain considerable immigrant populations, with more than 44.7 million immigrants residing within the country in 2018 [1]. Contemporarily, immigrants in the US are experiencing a rapidly changing policy landscape. The Trump administration’s restrictive policies including its “zero tolerance” immigration policy, the narrowing of asylum qualifications, and expansions of the “public charge” definition to include any individual who uses or is likely to use one public benefit, have dramatically narrowed and delayed the pathway to citizenship or permanent resident status and thus access to basic services for the estimated 10.5 million undocumented immigrants currently in the US [2].

Most recently, the astounding racial and ethnic disparities emerging between the victims of the 2020 SARS-CoV-2 pandemic are notable. Preliminary analyses from New York City have shown that Latinx individuals have the highest mortality (22.8%) of any reported ethnic group [3]. In fact, in 20 of 45 US states reporting ethnic data of COVID-19 cases, the proportion of cases among Latinx individuals is at least twice as high as what would be expected on the basis of population; in 11 of the 45 states with available data, the proportion of Latinx individuals infected is more than three times as high [4,5]. It is important to note, that these data do not distinguish between native, naturalized, noncitizen, or undocumented Latinx individuals; as can be expected, data on the morbidity and mortality of undocumented individuals are nearly impossible to gather. However, given that 44% of US immigrants report having Hispanic or Latinx origins, it can be taken as a certainty that the current pandemic has greatly and disproportionally affected immigrant communities [1]. Previously, the definition of “public charge” included Supplemental Security Income, Temporary Assistance for Needy Families, state general assistance programs, and long-term, publicly funded institutionalization. The current administration has added non-emergency federally funded Medicaid, the Supplemental Nutrition Program known colloquially as “food stamps,” Section 8 housing assistance, public housing, and state and local cash assistance. Of note, while the Trump Administration had suspended enforcement of the new public charge rule on July 29, 2020, over four months after the onset of the COVID-19 pandemic in the US, the law was reinstated as enforceable on September 11, 2020 [6]. As cases of COVID-19 continue to hit record highs across the US, the Trump Administration has yet to resuspend enforcement of this rule [7]. As such, immigrants in the US today are faced with the unprecedented disadvantage of having to navigate a global pandemic in the setting of an exceedingly hostile immigration climate.

To examine the historical effects anti-immigrant policies have had on health outcomes for communities they directly affect, we systematically selected and reviewed studies published after the Immigration Act of 1990 that investigated the adverse health effects that specific anti-immigrant policies had on immigrant communities in the US.

## Methods

This study was performed following the Preferred Reporting Items for Systematic Reviews and Meta-Analyses (PRISMA) statement [8]. In adherence to these guidelines, a protocol was registered in PROSPERO (registration #CRD42019138817).

### Search strategy

A medical librarian performed comprehensive searches to identify studies that evaluated local, state, or federal anti-immigrant laws and examined their effects on the health of immigrant communities in the US. We define anti-immigrant law as any law or policy at the local, state, or federal level that serves to restrict immigrant access to basic services, public benefits, or employment, or increase the threat of legal consequence or deportation. Studies that exclusively assessed the effects of positive or inclusive immigrant laws were excluded from analysis, as an analysis of the protective effects of positive immigrant laws was beyond the scope of our research question. However, studies that assessed both positive and punitive laws were eligible for inclusion. Searches were initially run on July 8, 2019, and updated on May 4 2020, in the following databases: Ovid MEDLINE^®^ (ALL 1946 to present); Ovid EMBASE (1974 to present); Cochrane Library (Wiley); Web of Science Core Collection (Clarivate Analytics); CINAHL (EBSCO); and Social Work Abstracts (Ovid). Search terms included all subject headings and associated keywords for the concepts of immigrants/migrants and law/policy in the US. Specific punitive legislation was also searched by name. The full search strategy for Ovid MEDLINE is available in Supplemental File 2. No language or article type restrictions were imposed.

### Study selection

**Fig 1:**
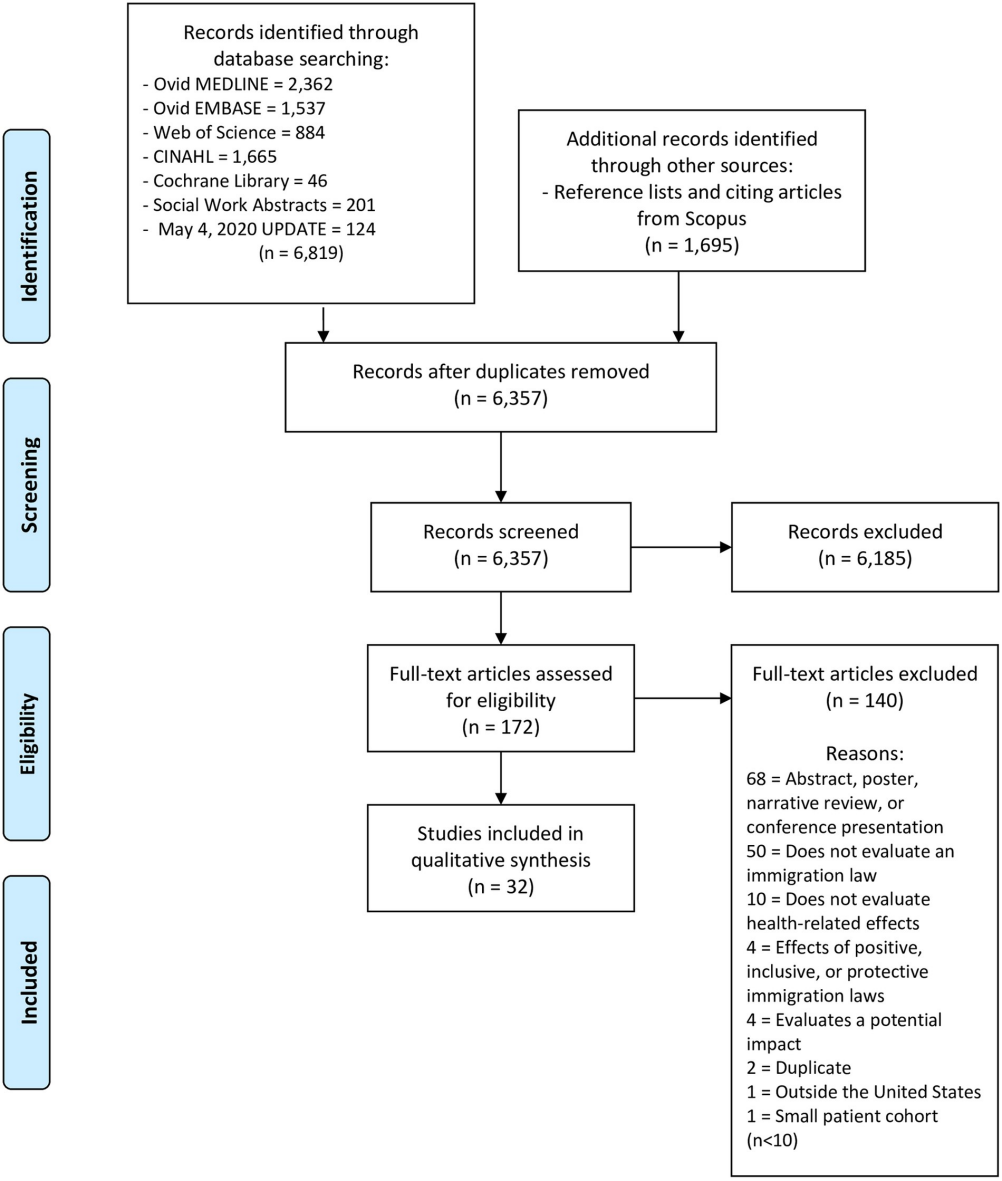
PRISMA flow diagram. Preferred reporting items for systematic reviews and meta-analyses (PRISMA) flow diagram outlining the process of study identification and selection. *From:* Moher D, Liberati A, Tetzlaff J, Altman DG, The PRISMA Group (2009). *P*referred *R*eporting *I*tems for *S*ystematic Reviews and *M*eta-*A*nalyses: The PRISMA Statement. PLoS Med 6(7): e1000097. doi:10.1371/journal.pmed1000097 **For more information, visit** www.prisma-statement.org.

After de-duplication, two independent reviewers screened 6,357 citations using Covidence systematic review software. Titles and abstracts were reviewed by two authors against pre-defined inclusion/exclusion criteria (Cohen’s kappa = 0.324). Studies that evaluated the health-related effects of a specific punitive immigrant policy in the US published after the year 1992 were considered for inclusion. Excluded articles were those that: (1) focused exclusively on the effects of positive, inclusive, or protective immigrant laws; (2) were outside the US; (3) had a small patient cohort (n<10); (4) evaluated a potential, rather than demonstrated, health impact; (5) did not study the effects of specific immigrant legislation or policy enactment; and (6) were published before 1992. Discrepancies were resolved by consensus. Due to lack of data to demonstrate impact, conference abstracts, posters, and presentations were also excluded. Full text publications were then obtained for 172 selected studies for a second round of eligibility screening (Cohen’s kappa = 0.533). Reference lists of included articles and articles citing included studies were pulled from Scopus (Elsevier) and also screened. The PRISMA flow diagram is available in Fig 1. Data were extracted by three reviewers independently with standardized forms in order to collect the following variables: study setting; population and participant demographics and baseline characteristics; details of the legislation; impact of legislation; follow-up time; follow-up data; study methodology. Due to data heterogeneity, no meta-analysis was performed. Study limitations were reported and risk of bias was assessed and noted.

## Results

A total of 32 articles were systematically selected for inclusion in this review (Table 1). These studies were qualitatively synthesized based upon four themes: (1) impact on healthcare utilization, (2) impact on women’s and children’s health, (3) impact on mental health services, and (4) impact on public health. See Table 2 for a summary of each policy referenced herein.

**Table 1:** Summary of studies evaluating the health effects of punitive immigrant policies.

Author (year)	Study Type	State or Region	Number and Description of Participants (mean age)	Legislation of Interest	Effect of Policy on Health	Limitations/Risk of Bias
Fenton (1996) [31]	Exploratory time-series analysis	CA	**Outpatient episodes**: Hispanics: 417 (32.9) Non-Hispanic Whites: 1,388 (34.5) **Crisis episodes**: Hispanics: 409 (32.4) Non-Hispanic Whites: 2,694 (33.2)	California Proposition 187	26% decrease in young (18–45) Hispanic outpatient mental health use; temporary increase in crisis service use	Results limited to San Francisco County; small sample size; no assessment of the clinical significance of decreased outpatient mental health service use
Marx (1996) [9]	Statistical sampling of healthcare utilization data	CA	**11/1-11/30 1993**: Walk Ins: 528 **11/1-11/30 1994**: Walk Ins: 395 **12/1/1993**: Cancelled and no-show: 1040 **12/1/1994**: Cancelled and no-show: 940	California Proposition 187	Significant decrease (p<0.001) of new patient walk-ins between data from November 1 to November 30 1993 and 1994	No distinction between Hispanic patients or non-White Hispanics; no distinction for immigration status; small sample size; one center study
Moss (1996) [19]	Small-scale, qualitative interview series	CA	56 interviews conducted from women >18 years old, having given birth after 9/1/94 or currently pregnant, and have emigrated no earlier than 1981	California Proposition 187	Perceptions of increased racism and discrimination in the healthcare setting; increased barriers to employment precluding use of private health care services; new fear of deportation	Small sample size; qualitative data; no follow-up on clinical implications or adverse health outcomes in study population
Fenton (1997) [10]	Survey (mailed questionnaire and phone interview)	CA	129 primary care clinics randomly selected from a sampling frame containing 446, representing each of California’s counties. Clinics in which >50% of visits were for non-primary care services, or >50% of patients had private health insurance were excluded	California Proposition 187	Although 65% of primary care directors perceived a decline in patient visits or were aware of a person who delayed care due to the law, no significant decrease in primary care clinic visits was found	Lack of adequate controlling for fluctuation in other clinic data trends; 34 of 58 California counties represented; monthly data points not frequent enough to detect short-term (2 week) decreases in clinic visits immediately following election
Loue (2000) [11]	Semi-structured interview series	OH	251 participants (40.8) 18/251 were undocumented 36.3% were from former USSR countries; 25.9% from Asia or Southeast Asia	Personal Responsibility and Work Opportunity Reconciliation Act; Illegal Immigration Reform and Immigration Reform and Immigrant Responsibility Act	Failed to find an adverse effect of the 1996 welfare and reform laws on immigrants’ ability to access medical care; many undocumented workers indicated they would delay or refrain from seeking care due to fear of immigration consequences	A large proportion (58.6%) of the cohort were permanent residents, refugees, or asylees and their usage of public services was undisrupted by the laws; small sample sizes of undocumented immigrants
Spetz (2000) [20]	Retrospective case-control study	CA	Los Angeles County birth certificate data analyzed from 419,327 foreign-born mothers with fewer than 12-years of education who do not have private health insurance compared against those from 179,664 US-born women	California Proposition 187	Reported a significant but small decline in the use of prenatal care (p<0.001), delayed commencement of prenatal care (p<0.001), and a lower number of visits (p<0.001) with respect to controls, with no detectable deterioration in birth outcomes in this population	Unable to exclude all documented immigrants from sample
Sun Hee Park (2000)	Telephone interview series	CA	99 “key informant” interviews from 76 different organizations; 41 safety-net providers, 35 immigrant health care advocates, and 25 government personnel Individuals selected across Los Angeles County, San Diego County, San Francisco Bay Area, and the Central Valley	Personal Responsibility and Work Opportunity Reconciliation Act; Illegal Immigration Reform and Immigration Reform and Immigrant Responsibility Act	Strong consistency that patients were afraid of applying for state coverage (Medi-Cal) and of receiving prenatal care without Medi-Cal coverage. There was fear about information sharing between the DHS and INS and the possibility of being labeled a “public charge,” the slow and confusing implementation of immigration reforms, and the eligibility process itself	Small sample size; incomplete sampling of the state’s counties; lack of direct patient interviews
Berk (2001) [12]	Multisite, structured interview series	TX; CA	973 adult undocumented immigrants interviewed as part of the Project HOPE Undocumented Immigrant Health Care Access Survey	Proposition 187; Personal Responsibility and Work Opportunity Reconciliation Act	39% of respondents reported fear of not receiving medical services due to their undocumented status; there was less fear in larger cities	Risk of nonresponse bias; study design based upon extracting data from immigrants living in predominantly immigrant communities
Loue (2005) [24]	Interview series	CA	157 women of Mexican ethnicity residing in San Diego County; sample included 51 US citizens; 40 women with permanent resident status prior to August 22, 1996; 10 women who received permanent resident status after PRWORA passage; and 56 women who were undocumented	Personal Responsibility and Work Opportunity Reconciliation Act; Illegal Immigration Reform and Immigration Reform and Immigrant Responsibility Act	No statistically significant difference was found between immigration statuses in the degree of difficulty experienced in obtaining gynecological or prenatal care or the quality of the care; undocumented status was associated with the level of fear experienced in obtaining medical care due to immigration status as well as rumors that health care would be unavailable due to immigration status	Small geographic sample
Lurie (2008) [25]	Retrospective difference-in-differences analysis	CA; TX; FL; NY; IL; NJ	**Permanent residents**: 2,459 **Non-permanent residents**: 795	Personal Responsibility and Work Opportunity Reconciliation Act	The proportion of uninsured children of non-permanent residents increased by 10% relative to those of permanent residents after the Welfare Act’s passage (1996 v. 2001); 17% of children of non-permanent residents lost Medicaid coverage despite their eligibility	Some immigrants were encouraged to change their status given the passage of the Welfare Act which complicates the “difference-in-differences” design
Angus (2010) [13]	Retrospective bivariate analysis	OR	**Records from the Family Planning Expansion Project-billed visits that occurred between May 2005 and April 2008**: 425,381 **Records billed for which reimbursement through the Project was not claimed**: 273,451	Deficit Reduction Act	Between May 2005 and April 2008, individuals seeking care through the Family Planning Expansion Project decreased by 33%, versus 10% for non-Family Planning Project Individuals (p<0.01); there was also a 47% decline in visits for clients under the age of 18, compared with 31% for those aged >18 (p<0.01)	Associative, not causal findings; unable to determine if care was delivered outside the network of Plan providers
Brabeck (2010) [32]	Survey (questionnaire administered at site of a community organization)	Multistate (northeast region)	132 individuals (36.7); 70.5% of participants were women; 38% acknowledged being undocumented	Antiterrorism and Effective Death Penalty Act; Illegal Immigration Reform and Immigration Reform and Immigrant Responsibility Act; PATRIOT Act	**Three hypotheses tested**: 1: Parent’s legal vulnerability significantly predicted impact on the family environment, accounting for 27.1% of the variance 2. Parent’s legal vulnerability significantly predicted the impact on child well-being, accounting for 30.6% of the variance 3. Parent’s legal vulnerability and impact on family environment were significant predictors of child well-being, accounting for 64.1% of variance	Risk of underreporting undocumented status; lack of appropriately validated instruments
Fountain (2011) [21]	Retrospective cohort study	CA	4,937,363 California birth records from 1992 to 2000; 16,681 were confirmed to have an autism diagnosis by 2006 (85% were successfully linked to birth records)	Proposition 187	Adjusted autism rates for Hispanic children from 1992–1996 was comparable to Non-Hispanic whites (OR 1.0 to 1.1); 1997–1998 (first cohorts after Proposition 187’s demise) saw 25–30% greater odds of autism; rate starts to decline back towards previous levels in 1999; data in two birth cohorts demonstrated 13% lower odds of Autism for Hispanic children during the Proposition 187 period; similar drop seen post-9/11, though not significant	DSM-IV expanded criteria for autism diagnosis in 1994; effect measures are indirect
Cleaveland (2012) [18]	Semi-structured interview series	VA	57 individuals (age range 18–77; 31/57 were male; 42/57 were aged 50 or younger)	Prince William County, VA “Rule of Law” Ordinance	Individuals reported high costs, language difference, and perceived indifference or hostility on the part of personnel in the wake of the “Rule of Law” ordinance	Small sample size; qualitative summary of data; data gathered was largely perceptive
Amuedo-Dorantes (2013) [14]	Analysis of cross-sectional probability survey data	Tijuana-San Diego Border Region	**Deported sample**: 947 (32); 9% from an E-Verify state **Voluntary Returnee sample**: 286 (32); 6% from an E-Verify state	Statewide enactment of E-Verify	Likelihood probability models show E-Verify mandates do not have a significant effect on obtaining health care services or government assistance, but do raise deportation fears and reduce interstate mobility among voluntary returnees; E-Verify mandates reduce the likelihood of returning to the US in the near future among deportees by 0.23 (–0.26 + 0.03); deportees residing in non-E-Verify states are 35% more likely to report intent to reenter, whereas deportees in E-Verify states are only 9% more likely	small sample size; limited geographic location; exclusion of unauthorized non-returnees
Beniflah (2013) [26]	Retrospective chart review	GA	In the two 4 month pre-HB periods (July-October 2009–2010), there were an average of 38,460 pediatric ED visits In the 4 month post-HB87 period (July-October 2011), there were 43,676 pediatric ED visits	HB87	There was a decrease in patients self-identifying as Hispanic post-HB87 (18.3% vs 17.1%, p<.01); the percentage of high acuity Hispanic patients increased from 14.3% to 16.3%; a larger percentage of Hispanic patients were admitted post-HB87 (10.2% vs 8.7%, p<.01); Hispanics were the only ethnic group to see a decrease in visits and increase in acuity	Retrospective single-center study; Hispanic identity used as a proxy measure for undocumented status
Anderson (2014) [37]	National telephone survey data data analysis	AZ	**Pre-SB1070 sample**: 4,740 respondents **Post-SB1070 sample**: 5,983 respondents	SB1070	Spanish-speaking Latinos have a lower predicted probability of excellent and very good health and higher predicted probability of good and fair/poor health (discrete change coefficient = 0.033), with a formal LR test producing significant results	Subjective primary measure of self-reported health; no separate analysis for undocumented individuals
Toomey (2014) [27]	quasi-experimental, prospective, closed-survey longitudinal interview series	AZ	204 dyads of adolescent mothers and mother figures from Arizona (ie. mother, grandmother, aunt) 142 adolescent mothers (16.85) 137 mother figures (41.35)	SB1070	Adolescents reported declines in use of public assistance (b = –0.51; OR = 0.60; 95% CI = 0.39, 0.92) and were less likely to take their baby to the doctor (b = –1.41; OR = 0.24; 95% CI = 0.08, 0.70); younger adolescents were also less likely to use preventive health care post-SB1070 with respect to older adolescents; mother figures were less likely to use public assistance after SB1070 if they were born in the United States and if their post–SB1070 interview was closer to the law’s enactment (b = –0.47;OR = 0.63;95%CI = 0.39, 0.99)	Self-reported outcomes; single large metropolitan area in Arizona
White (2014a) [39]	Retrospective chart review	AB	140,856 pediatric and adult patient records from September 1, 2010 to August 30, 2012 **Latino visits**: 20,524	HB56	Among Latino adults, total monthly visits were at least 20% lower relative to the same month in 2010, with an overall decrease of 30% relative to the previous year, and for all service types; non-Latino adults saw a 2% increase in visits in the period after the law was implemented; for total visits and 4/5 specific visit types, the mean monthly percent change differed significantly for Latinos vs non-Latinos (p<.01); there was a 48% decrease in immunizations for Latino adults and a 28% decrease for non-Latinos (p = .054)	No means of differentiating between documented and undocumented Latino individuals; EHR data did not include symptoms when presenting for care and thus some non-exempt services may have included exempt conditions, thereby underestimating the law’s effect
White (2014b) [22]	Coded interview series	AB	30 interviews (32) **Mexican origin**: 24/30 **Uninsured**: 29/30	HB56	Many participants expressed confusion over health care eligibility, delays in seeking care, increased use in home remedies, fear of deportation, concerns over health care costs, fear of driving due to potential for deportation, and incidences of discrimination and mistreatment by healthcare staff	qualitative study; small sample size; self-reported data
Flores (2015) [40]	Retrospective fixed effects regression analysis	PA	**Dependent variabl**e: County-level gun sales data from 2004 to 2011 **Independent variables**: formal consideration of county-level anti-immigrant ordinance between 2004–2011	County level anti-immigrant ordinances proposed up to 4 years before year of interest for study Anti-immigrant ordinances defined as measures that directly or indirectly target undocumented immigrants	Proposal of anti-immigrant ordinances associated with 6% increase in handgun sales in counties where ordinances were considered	Aggregate data inhibits identification of differentiating characteristics of individuals purchasing guns after proposal of ordinances; no direct link to health consequences, rather an implication of potential public health ramifications
Rhodes (2015) [29]	Retrospective analysis	NC	6 focus groups (n = 66); 17 individual interviews with self-identifying Hispanic or Latino persons, >18 years old, Spanish speaking, and living in the county where the interview took place	Section 287(g) of Immigration and Nationality Act; Secure Communities program	No significant difference in utilization of prenatal care before and after implementation of 287(g) Subjective reports of mistrust of healthcare workers, delay in seeking care, and inadequate care received among interviewees	Retrospective analysis of birth data; use of proxy measures to assess effects on undocumented individuals
Rubio-Hernandez (2016) [34]	Series of semi-structured interviews	AZ	54 Latino immigrant parents with children between 7–12 years old	SB1070	Emotional impact of anti-immigrant legislation characterized by concern and sense of responsibility, fear and hypervigilance, sadness and crying, and depression	Study solely informed by parent’s perceptions rather than children’s point of view
Joseph (2017) [16]	Interview series	MA	153 immigrants, health care professionals, immigrant and health advocacy organization employees in Boston, MA	MA healthcare reform, 2010 ACA	Interviewees across stakeholder groups feel that immigrants’ documentation status minimizes access to healthcare despite health care coverage	Small, nonrandom sample limits generalizability of results
Kline (2017) [15]	Interview series	GA	45 undocumented immigrants, 18 health providers, 9 staff from health-related NGOs, 4 nonclinical staff, 3 state agency workers, 3 state legislators, 2 nonhealth-related activist organization leaders	Immigration law enforcement in GA	Immigrant policing negatively affects immigrants’ health behaviors and sites for seeking health services	Small sample size; qualitative interview series
Potochnick (2017) [30]	Cross-sectional pre-post study using linear probability models and difference-in-difference analysis	Federal law	Pooled data from the Current Population Survey Food Supplemental Survey (CPS-FSS) 2004–2009; n = 3,307 non-citizen Mexican households with children; n = 4,710 Hispanic citizen households; n = 40,427 non-Hispanic White citizen households; n = 7,905 Black citizen households	Section 287(g) of Immigration and Nationality Act	287(g) associated with 10% increase in food insecurity risk for Mexican non-citizen households with children	Results may be reflective of the Great Recession; movement of immigrants into or out of metro areas may introduce bias; limitations in determining documentation status from CPS-FSS data
Gurrola (2018) [38]	Focus group interview series followed by combined deductive/inductive coding of data	CA	108 participants recruited from a predominantly Latino school district in Southern California Participants were majority women (90%), had an average of 3 children, lived in the US for more than 10 years (75%), had less than 9 years of education (71%), and primarily spoke Spanish (68%)	SB1070	Participants reported experiences of discrimination in their work environment, by children in school, accessing health services, in public spaces/everyday activities, interactions with public officials, and limited social interactions; discrimination adversely impacts immigrant economic stability (uncertainty, lack of opportunities), education (bullying), health care access (uninsured, differential treatment), and social/community context (limited interaction, fear of deportation)	Qualitative study; majority women (90%); inability to isolate undocumented immigrants
Santos (2018) [35]	Survey conducted at two time points and multivariate regression analysis	AZ	689 school youth self-identifying as Latina/o (12); 51.2% were female	SB1070	Males who reported awareness of SB1070 at time 1 reported lower levels of classroom regulatory behavior at time 2, with the simple slopes test revealing that the regression slope for males was significantly different from zero (t(490) = −2.54, p = .01)	Study conducted at one middle school where Latina/o group was the majority
Wang (2018) [33]	Retrospective difference-in-difference analysis	Federal law	Adults born in Latin American aged 18–60 in households with at least one noncitizen member or only noncitizen family members; responses obtained via National Health Interview Survey from 2000–2012. Sample size for self-reported health and mental health outcomes for foreign-born Hispanics with at least one noncitizen house member were 71,241 and 24,210. Sample size for self-reported health and mental health outcomes for foreign-born Hispanics with only noncitizen house members were 18,948 and 7,680. Sample size for self-reported health and mental health outcomes for US-born non-Hispanic Whites were 172, 185 and 75, 090	Section 287(g) of IIRIRA Secure Communities Program	Local law enforcement policies increased mental health distress and decreased self-reported health status among Latino immigrants living with noncitizen family members	Based on national data sources which do not disclose legal residence status of undocumented immigrants
Gómez (2019) [17]	Semi-structured interviews	AZ	43 participants from mixed-status households from the Tucson area who identified as the primary decision maker regarding healthcare; 84% were female and 51% were aged 35–49	SB1070	Barriers to accessing healthcare for Latino living in mixed households include complicated applications processes for coverage, fear and discrimination related to detention and/or deportation, wait times, and health literacy; promoters to care included affordability of care, location of services, experience with front-line staff and assistance with applications	Generalizability of the study may be limited due to geographic limits and since random sampling was not possible
Torche (2019) [28]	Retrospective difference-in-difference model analysis	AZ	Natality microdata was obtained for 1,444,541 Latina immigrant mothers, 1,504,561 US-Born Latina mothers, and 2,403,044 US-born Black and White mothers from the Centers for Disease Control and Prevention and the Arizona Department of Health Services from January 2007-December 2012	SB1070	For Latina immigrants, birth weight significantly declined by 15g (0.53 ounces) in Latina immigrants during July-December 2010 who were exposed to passage of the law during gestation; no significant reduction was observed before or after this period; no significant reduction in birth weight was observed for US-born Latinas or black and white women	Mechanisms causing decreased birth weight cannot be identified with nationwide aggregate data; spillover effects to US-born Latinas were also not observed
Roche (2020) [36]	Prospective survey	GA	547 adolescents were surveyed (12.8) with a 6-month follow-up retention rate of 81.5% (446/547) 136/547 adolescents (24.9%) had a family member detained or deported in the previous year	Attorney General “Priority” Directive; Attorney General “Zero Tolerance” Directive for “Improper Entry” into the United States	Family member detention or deportation was associated with higher odds of suicidal ideation (38/136 [27.9%] vs 66/411 [16.1%]; adjusted OR 2.37; 95% CI, 1.06–5.29), alcohol use (25/136 [18.4%] vs 30/411 [7.3%]; adjusted OR 2.98; 95% CI, 1.26–7.04), and clinical externalizing behaviors (31/136 [22.8%] vs 47/411 [11.4%]; adjusted OR 2.76; 95% CI, 1.11–6.84) at 6-month follow-up	self-reporting bias; short follow-up period

**Abbreviations**: CI = confidence interval; DHS = Department of Homeland Security; HB = house bill; INS = Immigration and Naturalization Service; SB = senate bill; OR = odds ratio; PRWORA = Personal Responsibility and Work Opportunity Reconciliation Act.

**Table 2:** Summary of relevant immigrant-related laws.

Law, Policy, Mandate, or Directive	Year Passed	Location	Relevant Provisions
Proposition 187	1994	California	Despite never fully going into effect, would have made undocumented immigrants ineligible for all non-emergent state-funded health care, and also require health care professionals treating suspected undocumented individuals to report to the INS
E-Verify Mandate	1996	Various	Initially established as part of the IIRIRA as a voluntary opt-in program run by the federal government; requires that employers submit information on a potential employee’s employment eligibility verify (I-9) form to be matched to government records
Personal Responsibility and Work Opportunity Reconciliation Act (“Welfare Act”)	1996	Federal law	Set strict provisions for those permitted to access non-emergent public benefits, and set a five-year lifetime limit to their use; lawful permanent residents, asylees, and refugees were deemed “qualified aliens” and remained covered under Medicaid; additionally, qualified aliens who entered the country after the date of the law’s enactment were subjected to a five-year bar on the receipt of benefits, including medical benefits, with asylees and refugees exempt again
Illegal Immigration Reform and Immigrant Responsibility Act (IIRIRA)	1996	Federal law	Allocated greater resources for border enforcement and wall construction, enacted civil penalties and potential jail time for illegal border passage, prohibited legal reentry for three years after deportation, and introduced means of expedited removal by which an INS officer rather than a judge may order the removal of an undocumented individual; also made undocumented individuals ineligible for Social Security benefits, in-state college tuition, and allowed states to deny driver’s licenses to such individuals as well, among other provisions and amendments
INA Section 287(g)	1996	Federal law	Provided local jurisdictions the option to participate in immigration enforcement in agreement with screening for immigration during regular policing operations (Task Force Enforcement) and in jails (Jail Enforcement)
Antiterrorism and Effective Death Penalty Act	1996	Federal law	Required the mandatory detention of non-citizens convicted of a wide variety of offenses including minor drug offenses; expanded the definition of “aggravated felons” that could be held in INS detention indefinitely
PATRIOT Act	2001	Federal law	Permitted indefinite detention for immigrants and non-citizens for offenses such as overstaying a visa, should their country refuse to repatriate them
Deficit Reduction Act	2005	Federal law	Mandated that states collect “satisfactory documentary evidence” of citizenship to qualify for Medicaid, altering the previous policy of citizenship attestation under penalty of perjury
Prince William County “Rule of Law” Ordinance	2007	Prime William County, Virginia ordinance	Mandated that police verify the immigration status of anyone suspected to be undocumented and barred undocumented individuals from accessing any social services not mandated by federal law; individuals without documentation were to be detained and remanded to ICE for deportation
Secure Communities Program	2008	Federal law	Created a network of data sharing allowing ICE access to information on immigrants held in jails who are deportable under US immigration law
Arizona SB1070 (Support Our Law Enforcement and Safe Neighborhoods Act)	2010	Arizona	Made residing and/or working in the US without legal permission a state crime. Required law enforcement officers to verify the legal status of all individuals who were arrested or detained; allowed law enforcement officers to arrest individuals without a warrant on the basis of probable cause of unlawful presence
Patient Protection and Affordable Care Act	2010	Federal law	Afforded lawfully present immigrants limited federal coverage; maintained the existing federal immigrant eligibility restrictions for Medicaid and CHIP except in states that chose otherwise; no federal coverage for undocumented immigrants
HB56 (Alabama Taxpayer and Citizenship Protection Act)	2011	Alabama	Required law enforcement officers to verify individual’s immigration status if there exists “reasonable suspicion” that they are undocumented; excluded undocumented individuals from all public benefits; directed schools to verify immigration status of all elementary and secondary students; schools can report students and parents presumed to be undocumented to the federal government; prohibited undocumented individuals from enrolling in any public postsecondary schooling or receiving financial aid; invalidated contracts between parties if one party is knowingly undocumented; established a Class C felony for an undocumented individual entering into a “business transaction” with a government agency; prohibited undocumented individuals from applying for or soliciting work; criminalized “harboring” or “transporting” undocumented individuals; created a state immigration police force
Georgia HB87 (Illegal Immigration Reform and Enforcement Act)	2011	Georgia	Required businesses in Georgia with >10 employees to verify that employees are eligible to work in the US legally; allowed police to verify immigration status of criminal suspects and detain persons suspected of being undocumented
Attorney General “Priority” Directive	2017	Federal directive	Directed federal prosecutors nationwide to prioritize certain immigration-related offenses for prosecution, namely improper entry into the US and illegal reentry after prior removal
Attorney General “Zero Tolerance” Directive for “Improper Entry” into the United States	2018	Federal directive	Directed federal prosecutors nationwide to implement a “zero tolerance” policy along the southwest US border, and to accept all improper entry cases to the extent possible

**Abbreviations**: ICE = Immigration and Customs Enforcement; INS = Immigration and Naturalization Service; CHIP = Children’s Health Insurance Program.

### 1. Impact on healthcare utilization

The 1994 passage of California’s Proposition 187 resulted in a significant decrease in new walk-ins (p<0.001) and no-show and patient cancelation rates (p = 0.01) in a study of ophthalmology clinic utilization, both of which only returned to baseline once Proposition 187 was stayed [9]. Additionally, while 65% of primary care directors perceived a decline in patient visits or were aware of a person who delayed care due to Proposition 187, their study did not find a significant decrease in primary care clinic visits in its wake [10].

In 1996, both the Personal Responsibility and Work Opportunity Reconciliation Act (“Welfare Act”) as well as the Illegal Immigration Reform and Immigrant Responsibility Act (IIRIRA) were passed; an interview series assessing their effects found that undocumented individuals consistently indicated they would refrain from or delay seeking medical treatment due to fears of immigration enforcement [11]. Another interview series of undocumented immigrants assessed the effects of Proposition 187 and the Welfare Act in both California and Texas, finding that 39% of respondents expressed fear of not receiving medical services due to immigration status. Interestingly, there was no significant decrease in those expressing fear in Texas with respect to California, which may suggest that Proposition 187’s effects extended beyond California’s border [12].

A 2010 study examining the effects of the Deficit Reduction Act on the utilization of Medicaid family planning services reported a 33% decrease in individuals seeking care through the Family Planning Expansion Project, versus 10% for non-Family Planning Project individuals (p<0.01), with a 47% decline in visits for clients aged <18, compared to a 31% decline for those aged >18 (p<0.01) [13].

A 2013 study assessing the effects of the E-Verify mandate on both voluntary returnees and deportees surveyed at the San Diego-Tijuana border crossing concluded that while E-Verify mandates did not have a significant effect on individuals’ ability to obtain healthcare services or government assistance, they did raise deportation fears and reduce interstate mobility among voluntary returnees [14].

A 2017 study investigating undocumented immigrants’ and healthcare providers’ perceptions of Georgia’s HB87, Section 287(g) of the Immigration and Nationality Act (INA), and the Secure Communities Program found that strict anti-immigrant policies increased fear and trauma among immigrants, which resulted in their refraining from or delaying care, restricting mobility, including for therapy or exercise, and seeking alternate care at small, private, fee-for-service clinics [15].

A 2017 Boston interview series of immigrants, healthcare professionals, and immigrant and health advocacy organization employees examining the relationship between municipal health reform and immigrant healthcare access revealed a consensus that immigrants’ documentation status hinders their access to healthcare regardless of health coverage status. Thus, even in states with inclusionary policies, federal exclusionary policies and national anti-immigrant sentiment can pose barriers to healthcare [16].

A study investigating how Latinx individuals living in mixed-status households navigate healthcare after SB1070’s passage found that over 50% of interviewees reported experiencing difficulty obtaining health coverage and endorsed the complexity of application as a major obstacle. Other barriers to care included discrimination and fear related to deportation and/or detainment while seeking public services, leading to severe delays or complete avoidance of care [17]. Similar concerns were raised in response to Prince William County, Virginia’s “Rule of Law” ordinance, with many Latinx individuals expressing concerns of high costs, language difference, and perceived indifference or hostility on the part of healthcare personnel, all of which suggest that these individuals are more inclined to forgo or delay seeking care [18].

### 2. Impact on women’s and children’s health

Three studies assessed the effect of Proposition 187 on Latinx women and children by using proxy markers for undocumented status such as foreign-born status, low educational attainment (<12 years), lack of private insurance, and residing in a county with a high proportion of undocumented immigrants [19–21]. In this population, the authors found a significant but small decline in the use of prenatal care (p<0.001), delayed commencement of prenatal care (p<0.001), and a lower number of visits (p<0.001) with respect to those of US-born women of similar educational attainment, with no significant difference in birth outcomes. These women expressed a sense of being “trapped” by their inability to seek publicly funded services, unjustly considered public charges, subject to increased racism and discrimination, and potentially subject to deportation. Combined with the effects of anti-immigrant legislation on the labor market, these women expressed an inability to enter the private market, while now ineligible for public assistance. Interestingly, after the staying of Proposition 187, autism diagnoses for Latinx-born children increased by 25–30%, suggesting an increase in healthcare utilization, which contrasts dramatically with the 13% decrease in autism diagnoses observed during the Proposition 187 period [19–21]. Similar concerns were reported by Latinx women in Birmingham in the wake of Alabama’s HB56, with many expressing confusion over healthcare eligibility, delays in seeking care, increased use in home remedies, fear of deportation, concerns over costs, fear of driving, and incidences of discrimination and mistreatment by healthcare staff [22].

Two studies examined the effects of the Welfare Act and IIRIRA on immigrant gynecologic and prenatal care. Interviews with safety-net providers, immigrant advocacy organizations, and government agencies revealed a consensus that patients were afraid to apply for public benefits and unable to utilize services in the absence of care, and credit fears of being labeled a “public charge,” confusion about the law implementation process, and eligibility requirements as exacerbating factors. While no significant difference was found in the attainment or quality of care when assessing patterns via residency status, the fear burden was significantly higher in undocumented individuals, who were more likely to report having heard rumors that healthcare was unavailable due to immigration status [23,24]. Although the Welfare Act had no direct effect on Medicaid eligibility for low-socioeconomic status children of non-permanent residents, it caused a significant “chilling effect,” with Medicaid coverage decreasing by 17% and levels of uninsured children rising by 10% with respect to those of permanent residents between 1996 and 2001 [25]. A similar effect was noted after Georgia’s HB87, with a significant decrease in Latinx pediatric patients presenting to the Emergency Department in the post-HB87 period (18.3% vs 17.1%, p<.01), an increase in the percentage of high acuity Latinx patients (14.3% to 16.3%), and a significant increase in Latinx patients admitted post-HB87 (10.2% vs. 8.7%, p<.01). Of note, Latinx was the only ethnic group to see a decrease in visits and increase in acuity in the study [26].

Two studies assessed the effects of SB1070 on Latinx mothers. The law’s passage was associated with declines in adolescent use of public assistance (b = –0.51;OR = 0.60; 95% CI = 0.39, 0.92) who were also were less likely to take their baby to the doctor (b = –1.41;OR = 0.24; 95% CI = 0.08, 0.70); compared with older adolescents, younger adolescents were less likely to use preventive healthcare after SB1070. Mother figures were also less likely to use public assistance after SB1070 if they were born in the US and if their post–SB1070 interview was closer to the law’s enactment (b = –0.47; OR = 0.63;95%CI = 0.39, 0.99). With regards to birth weight, a significant decline by 15 g (0.52 oz) was observed between July-December 2010, suggesting that these effects were linked to SB1070’s signing into law rather than implementation. No significant decreases in birth weight were observed in US-born Latinx, black, or white women [27,28].

While a 2015 study examining how implementation of the Secure Communities Program and section 287(g) of the INA affected prenatal care utilization among Latinx women in North Carolina did not find significant differences in care utilization before and after policy implementation, Latinx mothers were found to delay and receive inadequate care compared to non-Latinx mothers. Additionally, Latinx individuals reported intense mistrust and avoidance of healthcare services [29]. Potochnick and colleagues also looked at the effects of local-level immigration enforcement in a 2016 study analyzing the consequences of section 287(g) on food security of Latinx immigrant children, finding that enforcement of 287(g) was associated with a 10% increase in food insecurity in Mexican non-citizen households with children [30].

### 3. Impact on mental health

A 1996 study examining the effects of Proposition 187 on the use of mental health services in San Francisco reported a 26% decrease in Latinx persons aged 18 to 45 seeking outpatient mental health services, net of any weekly outpatient episodes or shared variance between young Latinx individuals and non-Hispanic whites. Moreover, there was a temporary increase in the use of crisis services by young Latinx individuals in the six weeks following Proposition 187’s passage [31].

A 2010 analysis examining the effect of detention and deportation on Latinx families and children in the wake of the Antiterrorism and Effective Death Penalty Act, the IIRIA, and the PATRIOT Act found that higher levels of legal vulnerability corresponded to a greater impact of detention or deportation on several critical aspects of their lives, including parent emotional well-being, ability to provide financially, relationships with children, children’s emotional well-being and academic performance, and ultimately, poor outcomes for children [32]. A study assessing the health and mental health effects of Section 287(g) of the INA and the Secure Communities Program on mixed-status households identified a causal relationship between local immigration enforcement policies and adverse effects on the health and mental health status of mixed-status Latinx households [33].

Three studies assessed the effects of anti-immigrant policies on the mental health of Latinx children, two of which focused on SB1070. These studies found that SB1070 had a negative emotional impact on children whose parents had been deported or who feared deportation, and ultimately support emotional trauma as a serious unintended consequence of anti-immigrant policies. Analysis of youth behavior in one Arizona middle school revealed that awareness of SB1070 may adversely impact academic adjustment and regulatory behavior in the classroom, which may in turn diminish education achievement; males who were aware of SB1070 reported lower regulatory behavior at a later time point [34,35]. An evaluation of the mental health effects of Attorney General Sessions’ 2017 and 2018 directives that dramatically increased deportation rates and intensified a policy of family separation on Latinx adolescents indicated that adolescents with a family member detained or deported had higher odds of suicidal ideation (38/136 [27.9%] vs 66/411 [16.1%]; adjusted OR 2.37; 95% CI, 1.06–5.29), alcohol use (25/136 [18.4%] vs 30/411 [7.3%]; adjusted OR 2.98; 95% CI, 1.26–7.04), and clinical externalizing behaviors, including aggression and rule breaking (31/136 [22.8%] vs 47/411 [11.4%]; adjusted OR 2.76; 95% CI, 1.11–6.84), at 6-month follow-up [36].

### 4. Impact on public health and daily life

Two studies found adverse effects of Arizona’s SB1070 on self-reported health and increased discrimination, respectively. Spanish-speaking Latinx individuals had a lower predicted probability of excellent and very good health and higher predicted probability of good and fair/poor health (discrete change coefficient of 0.033), with a formal LR test producing significant results. In an assessment of “spillover effects” of SB1070, study participants reported experiencing discrimination in environments including work, school, healthcare, and within their communities. Using a social determinants of health framework, these experiences adversely impact participant economic stability, education, health access, and limit social interactions [37,38].

A 2014 study evaluating changes in Latinx public healthcare service utilization prior to and following Alabama’s HB56 found that total monthly visits were at least 20% lower relative to the same month in the previous year, with an overall decrease of 30% relative to the previous year for all service types, including communicable diseases, sexually transmitted infections, and immunizations. For total visits and four of the five specific visit types, the mean monthly percent change differed significantly for Latinx adults *vs*. non-Latinx adults (p<0.01) [39].

A 2015 study examined the impact of proposed restrictionist immigration ordinances in 24 Pennsylvania counties on local gun ownership. The study included all ordinances proposed by elected officials that directly or indirectly targeted undocumented immigrants. Although none of the anti-immigrant measures was ultimately enacted, their proposal was associated with an increase in handgun sales in these counties, raising concern for a potential increase in gun violence [40].

## Discussion

Our findings demonstrate how specific anti-immigrant legislation negatively affects the communities they target. The US is unquestionably experiencing heightened anti-immigrant sentiment. For example, in the Latinx community a 2016 study found that 70% of Latinx individuals reported discrimination in their daily life, a dramatic increase from 30% reported in 2002–2003 [41]. Previously, it has been shown that an anti-immigrant climate is associated with worse health outcomes, poorer self-reported mental health, and increased discrimination in Latinx communities [41–45].

Previously, and predictably environments hostility towards immigrants have been shown to correlate with decreased or delayed healthcare utilization. In California, for example, a main predictor of not seeking medical care is undocumented status [46]. Even with basic health care services that remain covered, such as childhood immunization, the incongruous policy between one government agency aiming to persecute undocumented individuals as another encourages them to seek preventative healthcare services is highly problematic, particularly in the era of the “public charge” which penalizes non-emergent Medicaid usage. Resultant delays in medical diagnoses, linkage to treatment services, and continuation of care for communicable diseases pose a substantial individual and public health risk. Consider, for example, the 1980s Los Angeles measles outbreak, which was partly attributed to low vaccination uptake by the children of undocumented individuals [47,48]. At this time, Latinx two-year old children in California were 50% less likely to be up-to-date with measles immunization than their Non-Hispanic White counterparts, while Latinx children in families with a Mexican-born parent and child were 15 times more likely to underutilize healthcare and 43 times more likely to be unimmunized to measles than Latinx individuals with a US-born parent and child [49,50].

Even modest deterrents of healthcare uptake can have considerable consequences for public health. For example, a 1994 study by Asch *et al*. noted that, while only 6% of undocumented patients with active tuberculosis feared that seeking treatment might lead to trouble with immigration authorities, those individuals were almost four times as likely to delay seeking care for more than two months, a period likely to result in disease transmission [51]. Furthermore, it is important to acknowledge that undocumented immigrants are overwhelmingly excluded or discouraged from accessing care, and that new strictures only compound preexisting and longstanding barriers to access including lack of insurance, high mobility, and low health literacy, among others [49]. This conclusion provides considerable reason for concern that contemporary anti-immigrant legislation has more than likely exacerbated the current epidemic we face.

It is worth noting that some policies, while increasing fear and confusion among immigrant communities, had little or no demonstrable effect on healthcare utilization or outcomes [10,11,14,20,24,29]. Several authors credited the inability to select for undocumented individuals, and thus the inadvertent inclusion of permanent residents, refugees, and asylees in their studies as a potential yet important confounder that might underestimate the true harms of the evaluated laws given their continued protection and coverage despite restrictions placed upon undocumented individuals. Additionally, many authors credited the expansion of safety net programs and training of frontline healthcare staff with mitigating potentially devastating health outcomes for these communities. Indeed, particularly in the era of the new “public charge” rule, more information dissemination, engagement, and support at the community level is necessary to maximize health equity for immigrant communities.

## Limitations, conclusions, and future directions

Our study has several limitations. First, extensive data heterogeneity precluded meta-analysis. Second, while all immigrant communities were of interest and no restrictions were placed upon our search strategy regarding the race or ethnicity of subjects, the overwhelming majority of studies eligible for screening focused exclusively on Latinx communities, and for that reason our final manuscript retains the same focus as a function of the studies that were ultimately included. Additionally, as previously stated, we acknowledge that Latinx Americans are not all immigrants, and that this study fails to assess the adverse health effects of punitive laws on non-Latinx immigrant communities. Moreover, most studies found it logistically impossible to isolate undocumented individuals for analysis, using proxies such as zip code, level of educational attainment, or uninsured or Spanish-speaking status to estimate their risk. Thus, much of the above data may underestimate the true effects of these laws on undocumented communities.

In conclusion, our study shows that many punitive immigrant policies have decreased immigrant access to and utilization of basic healthcare services, while instilling fear, confusion, and anxiety in these communities. The federal government should preserve and expand access for undocumented individuals without threat of deportation to improve health outcomes for US citizens and noncitizens. Future research on this topic should attempt to analyze the effects of anti-immigrant legislation specifically on undocumented individuals to more accurately assess outcomes and provide stronger evidence to guide policy.

## Supporting information

S1 File PRISMA checklist. (DOC)

S2 File Full MEDLINE search strategy. (DOCX)

S3 File Preliminary search strategy. (DOCX)
