# Supplementary material for: The adverse health effects of punitive immigrant policies in the United States: A systematic review
Source: PLoS One. 2020 Dec 16;15(12):e0244054. doi: 10.1371/journal.pone.0244054 (PMC7744052; doi:10.1371/journal.pone.0244054)
Supplement: S2 File — (DOCX) [file pone.0244054.s002.docx]

**Ovid MEDLINE** (ALL – 1946 to present)

Searched on July 8, 2019; updated May 4, 2020

No language or publication type restrictions

| **Line #** | **Search** |
| --- | --- |
| 1 | **"Emigration and Immigration"/** |
| 2 | (immigration* or emigration* or migration* or in-migration* or out-migration* or border crossing*).tw. |
| 3 | **"Emigrants and Immigrants"/** |
| 4 | (immigrant* or emigrant* or foreigner or foreigners or alien or aliens).tw. |
| 5 | **Undocumented Immigrants/** |
| 6 | undocumented worker*.tw. |
| 7 | **"Transients and Migrants"/** |
| 8 | (transient* or migrant* or nonmigrant* or nomad*).tw. |
| 9 | or/1-8 |
| 10 | **Legislation as Topic/** |
| 11 | legislation jurisprudence.fs. |
| 12 | (legislation* or law or laws or jurisprudence or amendment or senate bill or court decision* or government regulation* or statute* or lawsuit* or litigation* or ordinance* or civil suit*).tw. |
| 13 | **Legislation, Dental/ or Legislation, Drug/ or Legislation, Hospital/ or Legislation, Medical/ or Legislation, Nursing/ or Legislation, Pharmacy/** |
| 14 | **Public Policy/** |
| 15 | (public policy or public policies or social policy or social policies or population policy or population policies or social protection or governmental policy or governmental policies or government policy or government policies or immigration policy or immigration policies or immigrant policy or immigrant policies or anti-immigration policy or anti-immigration policies or anti-immigrant policy or anti-immigrant policies).tw. |
| 16 | **Policy Making/** |
| 17 | (policy making or policy maker* or policy development* or policy analysis or policy analyses).tw. |
| 18 | **Health Policy/** |
| 19 | (health policy or health policies or health care policy or health care policies or healthcare policy or healthcare policies).tw. |
| 20 | **Health Care Reform/** |
| 21 | (health care reform* or healthcare reform*).tw. |
| 22 | ("proposition 187" or "Save our State Initiative" or "South Carolina SB 20" or "South Carolina senate bill 20" or "South Carolina's SB 20" or "South Carolina's senate bill 20" or "Taxpayer and Citizen Protection Act" or "Support Our Law Enforcement and Safe Neighborhoods Act" or "Arizona SB 1070" or "Arizona senate bill 1070" or "Arizona's SB 1070" or "Arizona's senate bill 1070" or "Alabama HB 56" or "Beason-Hammon Alabama Taxpayer and Citizen Protection Act" or "Personal Responsibility and Work Opportunity Reconciliation Act").tw. |
| 23 | or/10-22 |
| 24 | **exp United States/** |
| 25 | (United States or US or USA or "U.S." or "USA" or Alabama* or Alaska* or Arizona* or Arkansas* or California* or Connecticut* or Delaware* or Florida* or Georgia* or Hawaii* or Idaho* or Illinois* or Indiana* or Iowa* or Kansas* or Kentucky* or Louisiana* or Maine* or Maryland* or Massachusetts* or Michigan* or Minnesota* or Mississippi* or Missouri* or Montana* or Nebraska* or Nevada* or New Hampshire* or New Jersey* or New Mexico* or New York* or North Carolina* or North Dakota* or Ohio* or Oklahoma* or Oregon* or Pennsylvania* or Rhode Island* or South Carolina* or South Dakota* or Tennessee* or Texas* or Utah* or Vermont* or Virginia* or Washington* or West Virginia* or Wisconsin* or Wyoming* or Appalachian region* or Midatlantic region* or Mid-atlantic region* or New England*).tw. |
| 26 | 24 or 25 |
| 27 | 9 and 23 and 26 |
| 28 | limit 27 to yr="1992 -Current" |
