# Supplementary material for: The adverse health effects of punitive immigrant policies in the United States: A systematic review
Source: PLoS One. 2020 Dec 16;15(12):e0244054. doi: 10.1371/journal.pone.0244054 (PMC7744052; doi:10.1371/journal.pone.0244054)
Supplement: S3 File — (DOCX) [file pone.0244054.s003.docx]

(((((("1992/12/31"[Date - Completion] : "2019/06/15"[Date - Completion])) AND (health care access)) AND (immigration)) AND (undocumented immigrants)) AND (United states)) AND (law)
